# Amarogentin relieves cholestatic liver injury caused by ANIT in rats by regulating the FXR and Nrf2 pathways

**DOI:** 10.22038/ijbms.2025.87063.18815

**Published:** 2026

**Authors:** Wenxiang Wang, Wei Xiong, Jingxin Mao, Chunyu Chen

**Affiliations:** 1 Chongqing Three Gorges Medical College, Chongqing 404120, China; 2 Chongqing Key Laboratory of Development and Utilization of Genuine Medicinal Materials in Three Gorges Reservoir Area, Chongqing 404120, China; 3 Chongqing Medical and Pharmaceutical College, Chongqing 401331, China

**Keywords:** α-Naphthylisothiocyanate, Amarogentin, Cholestasis, FXR, Mechanism, Nrf2

## Abstract

**Objective(s):**

Cholestasis, a hepatic disorder characterized by impaired bile secretion, drives progressive liver damage, fibrosis, failure, and even death. This study explores how amarogentin (AG) ameliorates cholestatic liver injury in rats induced by α-naphthylisothiocyanate (ANIT).

**Materials and Methods:**

The bile flow rate, a visual indicator of the degree of intrahepatic cholestasis, was measured to assess the model’s success. Liver function was evaluated by analyzing the serum levels of enzymes (ALP, ALT, AST, TBIL, DBIL, and TBA), as well as indicators of oxidative damage (SOD, MDA, and GSH-Px), in the liver tissue, and by examining liver histopathology. Additionally, Western blot analysis was utilized to assess the protein levels of the FXR and Nrf2 signaling pathways in the liver tissue of cholestatic rats both before and after AG treatment, to understand the underlying protective mechanism.

**Results:**

AG was administered intragastrically to ANIT-treated cholestatic rats, which significantly decreased the plasma concentrations of AST, ALT, ALP, TBIL, DBIL, and TBA, and alleviated ANIT-induced liver injury. AG could also significantly improve the bile flow rate and suppress oxidative stress. Western blot analysis revealed that AG could enhance ANIT-induced cholestasis by modulating the anti-oxidative system via activation of the PI3K/Akt/Nrf2 pathway and by regulating bile acid metabolism.

**Conclusion:**

This study demonstrated that AG may mitigate ANIT-induced cholestatic liver damage by improving the bile flow rates, decreasing the concentrations of liver function markers and serum enzyme levels, enhancing liver histology, activating Nrf2 via the PI3K/Akt signaling pathway, and controlling bile acid transport.

## Introduction

The liver is essential for various metabolic processes. However, multiple factors present within the body can damage the liver (1, 2). Bile acids play a crucial role in the metabolism of fats and fat-soluble vitamins, as well as in maintaining cholesterol homeostasis. Bile acids are mainly produced and broken down in the liver. They are then carried by transporters present on the basal and hepatic membranes of liver cells. A smooth flow of bile acids is essential for optimal health. Any obstruction of bile acid flow has serious ramifications. For example, intrahepatic cholestasis results when bile production, absorption, and circulation are adversely impacted by various factors (3). Intrahepatic cholestasis is characterized by high cholesterol levels in the body, itching and skin discoloration, and elevated levels of bilirubin and liver enzymes in bodily fluids, such as blood and urine (4). If not treated and managed in time, cholestasis may progress to liver inflammation, damaged liver cells (including portal myofibroblasts and hepatic stellate cells), and biliary fibrosis and/or cirrhosis in the worst cases. (5) Hence, cholestasis is a significant burden on both patients and society (6, 7).

The currently available treatments of choice for cholestasis are limited to the use of corticosteroids, budesonide, fibrates, and ursodeoxycholic acid (UDCA) (8). However, their effectiveness remains unsatisfactory. UDCA, a bile acid produced by intestinal bacteria, is currently employed as the primary medication for treating various cholestatic conditions. Unfortunately, it has a very low success rate and is unable to stop the advancement of cholestasis to cirrhosis and ultimately liver failure (9). Another medication frequently used to manage and treat cholestasis is a glucocorticoid. However, its prolonged use leads to multiple adverse effects. In addition, its discontinuation has a rebound effect (10). Therefore, it is essential to develop suitable medications and therapies for treating and managing cholestasis. FXR, or Farnesoid X receptor, belongs to the nuclear receptor superfamily of proteins. After being stimulated by bile acids, it can regulate the production, movement, release, and uptake of bile acids, facilitating numerous pathways that ultimately improve intrahepatic cholestasis (11). Therefore, FXR is attracting increasing interest as a potential pharmaceutical for cholestasis. FXR functions in a network, with various upstream signals regulating molecules downstream (12-14). Several potent FXR agonists, including PX20606 (PX-102), fexaramine, LMB763, tropifexor (LJN452), and GS9674, are currently in the clinical development stage and poised for widespread use. Nuclear factor erythroid 2-related factor 2 (Nrf2), a crucial transcription factor controlling oxidative and toxic stress, enhances the production of glutathione and controls the activity of bile acid transporters (15, 16). Therefore, it is capable of ameliorating the symptoms of liver cholestasis, and hence, it is attracting increasing attention for the treatment and management of cholestatic liver damage. 

Traditional Chinese medicine (TCM) has a long history of being used for various liver disorders, including intrahepatic cholestasis. In addition to offering numerous therapeutic benefits, TCM therapy provides several other advantages, including reduced toxicity and fewer side effects (17, 18). For example, the iridoid glycosides sweroside and swertiamarin, which are active TCM compounds, can provide protection against α-naphthylisothiocyanate-induced cholestasis by stimulating FXR and promoting the excretion of bile acids (19). 

**Figure 1:**
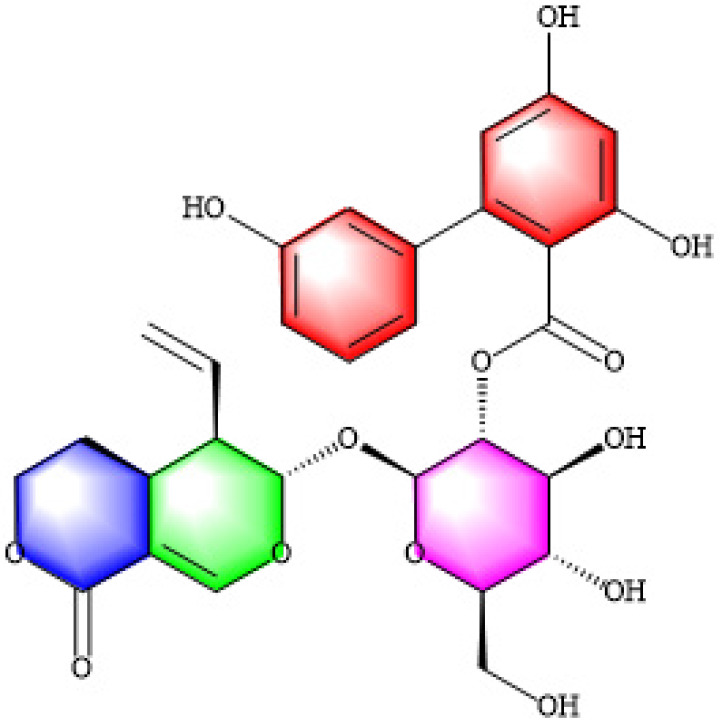
Chemical structure of amarogentin (AG, CAS number: 21018-84-8)

Secoiridoid glycosides exhibit various biological properties, such as anti-oxidative, antineoplastic, and antidiabetic epreguffects. Amarogentin (AG, Figure 1) is usually extracted from *Swertia davidii Franch*. Its properties are similar to those of sweroside and swertiamarin, and hence, it can exhibit an anti-cholestasis effect. Recent research has shown that AG can prevent liver cancer by controlling the G1/S cell cycle checkpoint kinase and promoting apoptosis during the early stages of dysplasia(20). Previous studies have demonstrated the liver-protective benefits of AG (21). Nevertheless, the particular mechanism through which AG protects the liver is still not fully understood. The present study assesses the protective impact of the ethanol extract of AG on ANIT-induced liver damage and bile duct obstruction in rodents. The levels of the enzymes and components in the serum and liver are also measured to assess liver function in different treatment groups. This study uses protein levels to measure the expression of bile metabolism-related transporters and oxidative stress-related factors in order to reveal the potential mechanisms through which AG exerts its protective effect against ANIT-induced cholestasis.

## Materials and Methods

***Materials***

ANIT (at least 98% purity) was obtained from Sigma-Aldrich (St. Louis, USA). Amarorgentin (at least 98% purity) was procured from Chengdu Desite Biotechnology Co., Ltd (Chengdu, China). UDCA was purchased from Losan Pharma GmbH (Freiburg, Germany). Commercial kits for direct bilirubin (DBIL), alkaline phosphatase (ALP), alanine aminotransferase (ALT), aspartate transaminase (AST), total bilirubin (TBIL), total bile acid (TBA), GSH, superoxide dismutase (SOD), and malondialdehyde (MDA) were provided by Nanjing Jiancheng Biotechnology (Nanjing, China). Antibodies against MRP2, BSEP, CYP7A1, NCTP, TGR5, p-PI3K, p-Akt, and FGF15 were acquired from Affinity Biosciences (Cincinnati, Ohio, USA). Antibodies against FXR, SHP, FGFR4, Nrf2, GCLc, GCLm, and GAPDH were acquired from Abways Technology (Shanghai, China). All other chemicals were procured from commercial sources and were of analytical grade.

***Animals and the experimental design***

A total of 48 male Wistar rats weighing between 220 and 240 g were acquired from Hunan Slake Jingda Experimental Animal Co., Ltd. (China) [permission reference SCXK (Xiang) 2019-0004]. The animals were kept in a chamber under controlled temperatures (25 ± 2 °C) and a 12-hr light/dark cycle. They were given unrestricted access to regular food and tap water. The rats were given a week to adjust before the experiment was started. The animal study was reviewed and approved by the Animal Ethics Committee of Chongqing Three Gorges Medical College. The experiments were conducted in compliance with the Guidelines for the Care and Use of Laboratory Animals (Approval No. SYYZ-A-2212-0005). 

**Figure 2:**
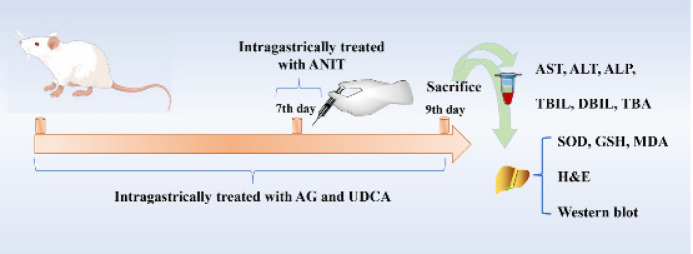
Workflow of amarogentin relieving cholestatic liver injury caused by ANIT in rats

All the animals were divided into the following six groups with eight animals each: a control group, a model group treated with ANIT, a positive group receiving UDCA at a dose of 60 mg·kg^-1^, and AG-L, AG-M, and AG-H groups administered AG intragastrically once a day at low (25 mg·kg^−1^), middle (50 mg·kg^−1^), and high (100 mg·kg^−1^) doses (22), respectively, for 7 days. Both the model and control groups were intragastrically administered with equal volumes of saline. In addition, ANIT dissolved in olive oil was administered intragastrically to each group at a dosage of 60 mg/kg (23), except for the control group that received an equivalent amount of intragastric olive oil without ANIT. All the rats were fasted for 12 hr before being sacrificed. The animals were sedated intraperitoneally with 2% sodium pentobarbital (0.2 ml/100 g) 48 hr after receiving ANIT. Serum enzymes were measured from the blood samples taken from the abdominal aorta. The liver was dislodged by using an aseptic apparatus. A part of the right large lobe was stored in 10% formalin for histological evaluation. The other samples were stored at -80 ℃ for the future assessment of hepatic proteins (Figure 2). 

***Sample collection and preparation***

The blood samples were immediately centrifuged for 15 min (4500×g, 4 °C). Prior to analyzing the biochemical markers, the serum samples were subjected to hemolysis, sterilized, and stored at -80 °C. A portion of the liver tissue was preserved in 4% paraformaldehyde for histopathological analysis, while the ileum and the remaining liver were preserved at -80 °C. 

***Analysis of bile flow rate***

Bile samples were collected every 30 min over a 1.5-hr period in pre-measured plastic tubes. The weight of each sample relative to the body weight of the rat was calculated using the following formula to determine the rate of bile flow: 

Bile flow μl=m2-m11000

Bile flow rate μlmin/100 g boy weight=(m2-m1)×100M×90

where m1 is the weight of polyethylene, m2 is the weight of polyethylene in the bile specimen. M is the body weight of the rat. Bile acid density is expressed in g·ml^-1^ (24). 

***Biochemical determination and histological analysis of serum***

The ALT, AST, ALP, TBIL, TBA, DBIL, GSH, MDA, and SOD levels in serum or liver tissue were assessed following the guidelines provided by the manufacturer. The liver samples were removed from the 4% paraformaldehyde solution, embedded in paraffin, and then cut into 5-μm slices. These slices were stained using H&E and examined under a Leica DMI8 light microscope (Wetzlar, Germany) at a magnification of 200×. 

***Western blot***

The liver tissue and the ileum tissue (50 mg) were homogenized in a protein lysis buffer containing 1 mM of PMSF. The mixtures were cooled on ice for 60 min and then spun at 12,000 rpm for 25 min at 4 °C. The protein levels were measured using a BCA protein assay kit (Beyotime, China). Next, 30 μg of protein per lane was separated using 8–12% of SDS-PAGE gel at 80 V for 30 min and 120 V for one hour. The proteins were then transferred to a nitrocellulose membrane. After blocking with 5% BSA, the membranes were incubated overnight at 4 ℃ with the antibodies against FXR, CYP7A1, MRP2, BSEP, NTCP, TGR5, SHP, and FGFR4 (diluted 1:1000), Nrf2, p-PI3K, p-Akt, GCLc, and GCLm (diluted 1:1200), as well as GAPDH (1:5000). The membranes were washed five times for 10 min each with phosphate buffer saline tween-20 (PBST) on the following day. After incubating the membranes with secondary antibodies conjugated with horseradish peroxidase, they were washed four times for 10 min each with PBST. The protein bands were identified using an advanced chemiluminescence detection system (Bio-Rad, USA). GAPDH was used as the internal control. 

***Molecular docking analysis***

The crystal structures of FXR and Nrf2 were obtained from the RCSB Protein Data Bank database (https://www.rcsb.org/). 2D configurations of AG, obtained from the PubChem database (https//pubchem.ncbi.nlm.nih.gov), were imported into Maestro 11.1 for molecular docking analysis. The OPLS_2005 force field was employed to minimize the ligand energy. The binding pocket coordinates were determined using the protoligand of the crystal structure as the center and a 1-nm box (10 Å). Other system parameters were set to default values. Molecular docking was performed using the ligand docking module with standard precision. The techniques employed in his study have already been outlined elsewhere (25). 

***Statistical analysis***

The data and graphs were generated using the GraphPad Prism 8.0 software (San Diego, USA). The data are presented as the mean plus the standard deviation (SD). A one-way ANOVA test was conducted to determine variances among the group averages. A statistically significant difference was determined at *P*≤0.05, while a highly significant difference was observed at *P*≤0.01. 

## Results

***Effects of AG on body weight, liver weight, and liver index***

**Figure 3:**
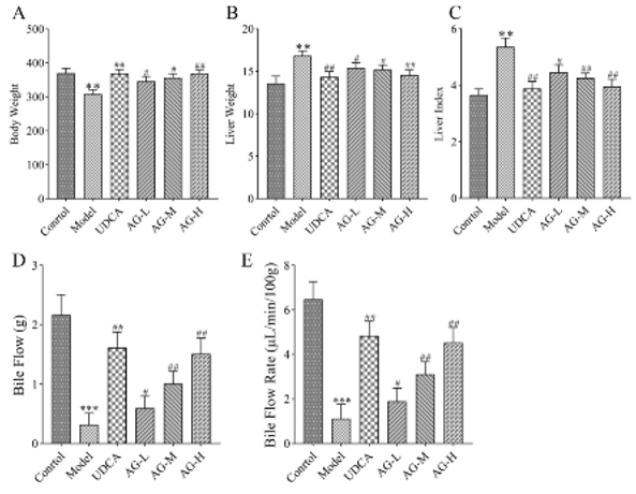
Effects of amarogentin (AG) on rat body weight (A), liver weight (B), liver index (C), bile flow (D), and bile flow rate (E)

The body weight, liver weight, and liver index (liver weight/body weight, %) are given in Fig. 3. The liver index was significantly higher in the ANIT group than it was in the control group (*P*<0.01). In contrast, the liver index of the AG group rats was much lower than that of the ANIT group (*P*<0.05, *P*<0.01, Figures 3A-C).

***Effects of AG on bile flow rate***

Bile flow obstruction is crucial for ANIT to induce cholestasis. In the model group, both the flow volume and flow rate of bile were significantly lower than those of the control group (*P*<0.01, Figures 3D-E). Cholestatic liver injury occurs when toxic bile acids build up due to the disruption of bile flow. This condition can be treated by restoring bile flow to a continuous state. In our study, we found that the rate of bile flow increased under moderate and high doses of AG, suggesting a positive effect of AG on cholestasis.

***Effects of AG on the serum levels of hepatic injury markers***

**Figure 4:**
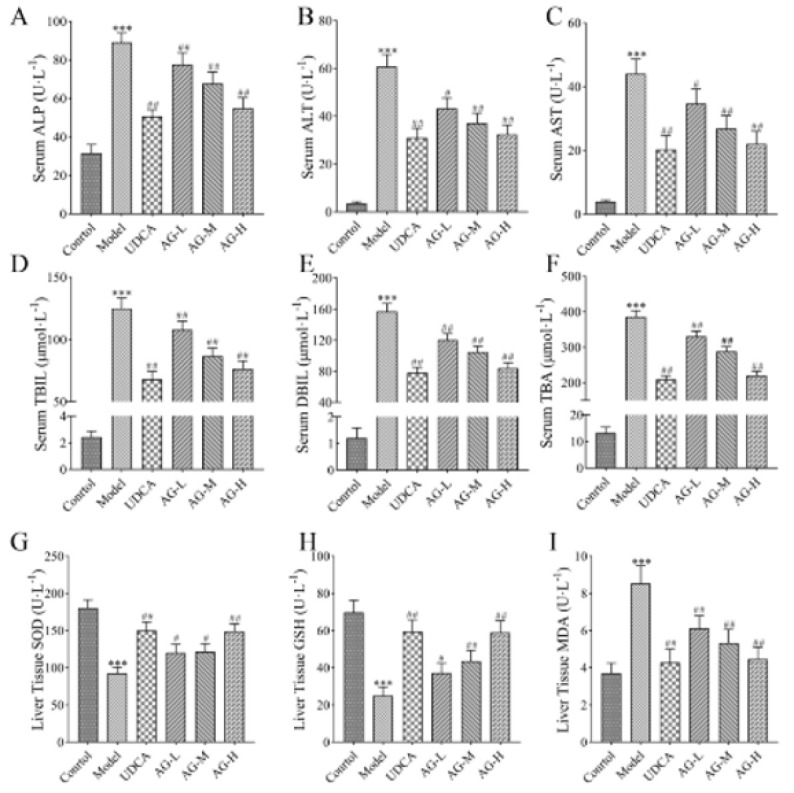
Effects of amarogentin (AG) on hematological parameters and oxidative stress indicators of rats

The hepatoprotective effects of AG were assessed by measuring the serum levels of liver injury indicators (AST, ALT, ALP, TBIL, DBIL, and TBA). As shown in Figure 4, the ANIT treatment resulted in a significant increase in the levels of these markers (*P*<0.001), indicating that the rats administered ANIT experienced liver damage. Both treatments with AG decreased the increase in the levels of hepatic biochemical indicators. High-dose AG treatment (100 mg/kg) provided superior protection against liver injury caused by ANIT, as evidenced by the marked reduction in the serum levels of AST, ALT, and ALP (*P*<0.001, Figures 4A-C). Even when only a small amount of AG (25 mg/kg) was used, a significant decrease was noted in the serum levels of the liver injury indicators (*P*<0.05, *P*<0.01).

***Effects of AG on the hepatic levels of SOD, GSH, and MDA***

Commercial assay kits were used to analyze the hepatic levels of SOD, GSH, and MDA, which serve as indicators of oxidative stress in liver tissue. The administration of ANIT notably increased the hepatic levels of MDA, while the administration of AG remarkably decreased them at different doses (Figures 4G-I). The ANIT group also exhibited a notable decrease in SOD and GSH levels compared to the control group. In contrast, the AG treatment elevated the concentrations of SOD and GSH.

***Histopathological evaluation of the liver tissue***

Histological assessment of the liver demonstrated the direct benefits of AG in preventing ANIT-induced cholestasis. The liver tissue of the control group exhibited a typical structure, with no unusual morphological alterations (Figure 5A). Conversely, in the ANIT group (Figure 5B), the liver tissue had common pathological alterations, including disrupted cellular boundaries, inflammatory cell infiltration in the portal region, and necrotic and interlobular duct epithelial injuries. Compared to the ANIT-treated group, the AG-treated group (Figures 5D-F) exhibited a notable enhancement in histological injury, aligning with the results of the serological examinations.

***Impact of AG on the regulation of bile acid balance in the liver tissue***

Western blot analysis was conducted to quantify the transporters and metabolic enzymes responsible for maintaining bile acid homeostasis in the rat liver. As shown in Figure 6, ANIT notably decreased the levels of FXR, MRP2, BSEP, NTCP, TGR5, and SHP proteins. However, this effect was significantly reversed by the AG treatment when compared to the control group. ANIT significantly increased the levels of CYP7A1 and FGFR4 proteins, whereas AG significantly decreased their expression.

***Impact of AG on the expression of oxidative stress-related factors in liver tissue***

We investigated the role of AG in cholestasis in an attempt to explore the oxidative stress associated with the PI3K/Akt/Nrf2 pathway. As shown in Figure 7, while analyzing the impact of AG on the ANIT-induced expression of GCLc and GCLm proteins in the liver, we observed that AG led to an increase in the expression of GCLc and GCLm. This increase suggests that AG may also elevate the GSH levels, which could help shield cells from oxidative harm. To investigate the impact of PI3K/Akt and Nrf2 on ANIT-induced cholestasis, we analyzed how paeoniflorin affected the expression of the p-PI3K, p-Akt, and Nrf2 genes in the liver. As shown in Figure 7, AG can enhance the protein levels of p-PI3K and p-Akt and also facilitate the translocation of Nrf2 to the nucleus.

***Molecular docking results***

As stated before, Maestro 11.1, a professional software program, was utilized to analyze active components and closely linked targets. We conducted molecular docking visualization and found that AG successfully penetrated the active sites of the main proteins (FXR and Nrf2) and formed interactions with particular amino acid residues through hydrogen bonding and protein interactions. AG formed two hydrogen bonds with the ASN287 and TYR373 residues of the FXR protein (Figure 8A). The Nrf2 protein formed seven hydrogen bonds with VAL512, VAL606, VAL369, ARG326, VAL561, and THR560 residues (Figure 8B). The functional activity of a protein typically depends on the presence of a limited quantity of functional residues. However, the determination of functional residues is often complicated by complex conservation patterns.

## Discussion

The use of synergistic components can explain the therapeutic potential of TCM treatment regimens. Similar to sweroside and swertiamarin, amarogentin, which may exhibit a protective effect against cholestasis, is also typically extracted from *Swertia davidii Franch*. We tested the hepatoprotective properties of AG in rats with ANIT-induced hepatic injuries. Additional studies are needed to validate the effectiveness of AG in treating liver damage caused by cholestasis, as indicated by the aforementioned results.

Cholestasis is characterized by an increase in related serological indices (26). The clinical manifestations of hepatic cholestasis can be identified by analyzing the serum levels of TBIL, DBIL, ALP, AST, ALT, and TBA. Hepatocytes primarily produce AST and ALT, which permeate the blood when ANIT damages the hepatocyte membranes. Moreover, liver diseases are typically diagnosed based on abnormal levels of AST and ALT. The degree of damage to hepatocytes has been speculated to correlate with the levels of AST and ALT aminotransferases. ALP is a serum enzyme that can provide information about the degree of damage to the biliary tract. Therefore, it is essential to determine its serum levels to diagnose biliary diseases. Increased levels of ALP are associated with intrahepatic cholestasis, obstructions of the bile ducts, and capillary cholangitis (27). For hepatocytes to function properly, it is necessary to maintain bile acid homeostasis. Obstruction of the bile duct and injury to liver cells disrupt the regulation of bile acids, leading to increased levels of total bile acids in the bloodstream. Notably, the detection of blood TBA levels is considered a sensitive test for liver function. Disorders of bilirubin metabolism are characterized by the accumulation of bilirubin in the blood. Thus, TBIL and DBIL are effective indicators of hepatic bilirubin metabolism (28). We found that AG reversed the ANIT-induced increase in serum concentrations of TBA, TBIL, DBIL, ALP, AST, and ALT, suggesting that AG ameliorated liver damage by repairing the blockage of bile ducts within the liver and reducing hepatocyte membrane permeability.

Cholestasis is also considered a typical clinical manifestation of hepatic diseases. Without timely intervention, cholestasis can progress to liver fibrosis and cirrhosis. The origin of cholestatic injury is complex, often resulting from disturbances in bile acid metabolism and circulation, hepatic accumulation of bile acids, and oxidative stress. Therefore, reducing the synthesis of bile acids, increasing their metabolism, restoring their flow, and reducing oxidative stress injury are widely studied as helpful treatment strategies. Currently, cholestasis has no effective and definite cure (29). Therefore, it is necessary to develop novel therapeutics for regulating bile acid metabolism. FXR and Nrf2 are two crucial factors influencing the transporters and anabolic enzymes of bile acids. Most studies on anti-cholestatic strategies have used only one factor, either FXR or Nrf2 (30, 31), despite both FXR and Nrf2 being crucial for controlling bile acid metabolism and its transport system. Nrf2 enhances the transcription of FXR and the subsequent gene expression, which in turn can trigger Nrf2 and its response to oxidative stress. Hence, we proposed that their interactions collectively control the activity of enzymes responsible for the synthesis and transport of bile acids, ultimately influencing their metabolic equilibrium. Therefore, we explored the protective properties of AG on cholestasis by focusing on the FXR and Nrf2 pathways (Figure 9).

FXR influences the production, breakdown, and movement of bile acids (32). It suppresses the production of bile acids by acting as a transcriptional inhibitor of the small heterodimer partner (SHP) through a feedback loop. Bile acids increase the expression of FXR, thereby activating the SHP transcription. SHP then interacts directly with LRH-1, a key regulator of bile acid production in the liver. SHP inhibits LRH-1, thereby inhibiting CYP7A1 (33, 34). Additionally, FXR regulates the hepatic concentrations of bile acids by influencing the transcription of proteins involved in their absorption and release. It enhances the activity of the bile salt export pump (BSEP) and multidrug resistance-associated protein 2 (MRP2), leading to increased bile excretion from the gallbladder and ultimately lowering its concentration in the liver (35). At the same time, FXR also inhibits the sodium taurocholate co-transporting polypeptide (NTCP) on the basolateral membrane, which binds with the bile acids reabsorbed from the intestine. Notably, FXR suppresses the reabsorption of renal proteins, thereby hindering the uptake of bile acids in the portal vein (36). The activation of FXR by the bile acids increases the transcription of fibroblast growth factor (FGF15 in rats and FGF19 in humans) in the intestinal tract. Once FXR reaches the liver, it binds to fibroblast growth factor receptor 4 (FGFR-4), which inhibits CYP7A1 transcription and reduces bile acid production (37). TGR5 is a widely expressed G-protein-coupled receptor in mammals (38). Bile acids maintain metabolic homeostasis and regulate lipid and glucose metabolism through the TGR5 receptor (39). Furthermore, TGR5 has been reported to modulate liver enzymes that synthesize bile acids (40).

UDCA is the only medication approved for treating cholestasis. It protects hepatic membranes from oxidative stress by increasing the cellular levels of both GSH and GCL (41). We evaluated the effects of AG on GSH and the relevant enzymes involved in its synthesis. Our findings indicate that AG increased the GSH levels and promoted the expression of GCLc and GCLm. Hence, it can be suggested that AG protects the liver from oxidative stress resulting from ANIT-induced cholestasis. In response to oxidative stress, the Nrf2 transcription factor controls the expression of GCL and other genes that help combat oxidative stress (42). Furthermore, Nrf2 translocation into the nucleus is regulated by PI3K (43). An increase in the synthesis of GSH can promote Nrf2 activity through the PI3K/Akt-dependent pathway, thus promoting the effect of downstream anti-oxidant target genes (44). Additionally, we investigated whether PI3K/Akt/Nrf2 signaling regulated GSH synthesis and whether AG up-regulated the GSH concentration through similar mechanisms. We observed that AG stimulated Akt phosphorylation, indicating that the PI3K/Akt pathway increased the GSH levels. In the ANIT-treated rats, AG increased the Nrf2 levels in the nucleus. It also ameliorated ANIT-induced cholestasis by up-regulating Nrf2, thereby regulating FXR and positively influencing the PI3k/Akt/Nrf2 signaling pathway.

Data (n = 8) are depicted as mean ± standard deviation. *** *P*<0.001 vs controls; # *P*<0.05, ## *P*<0.01 vs the model.

The levels of alkaline phosphatase (ALP, A), alanine aminotransferase (ALT, B), aspartate transaminase (AST, C), total bilirubin (TBIL, D), direct bilirubin (DBIL, E), total bile acid (TBA, F), superoxide dismutase (SOD, G), glutathione (GSH, H), and malondialdehyde (MDA, I) of the sample. Data (n = 8) are depicted as mean ± standard deviation. *** P<0.001 vs controls; # *P*<0.05, ## *P*<0.01 vs the model.

**Figure:**
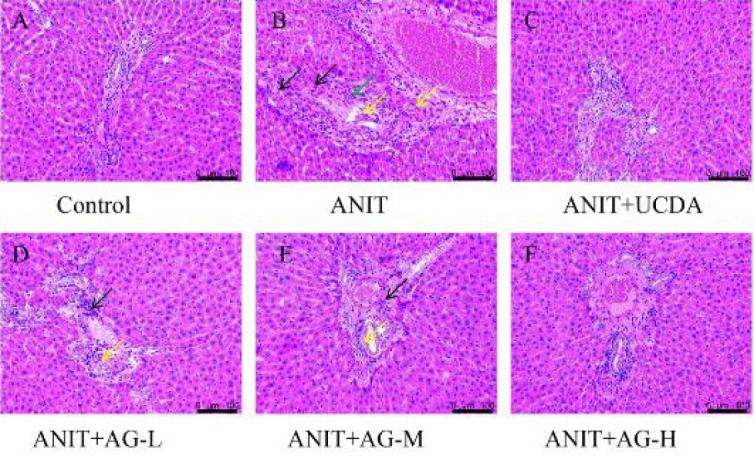


**Figure:**
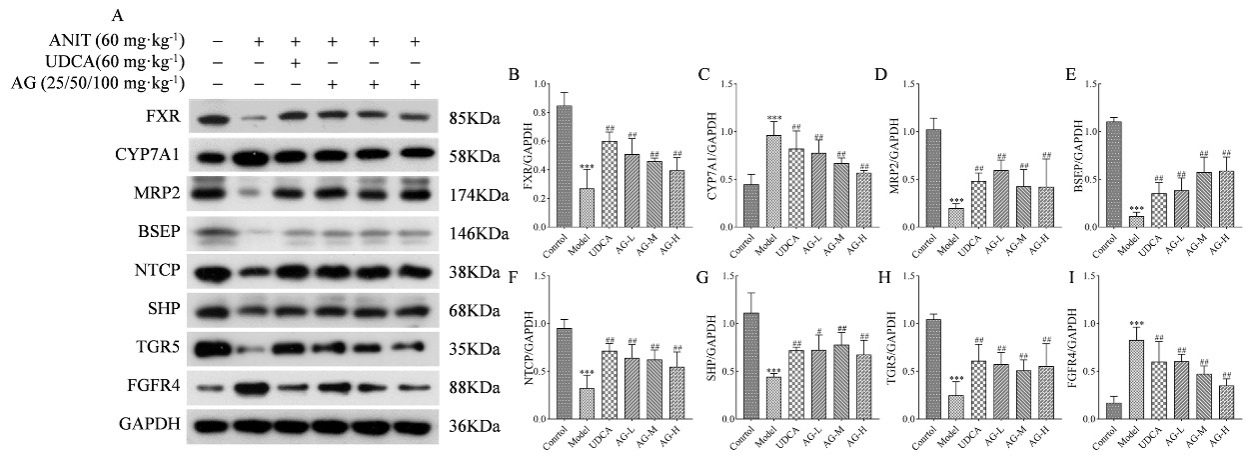


**Figure:**
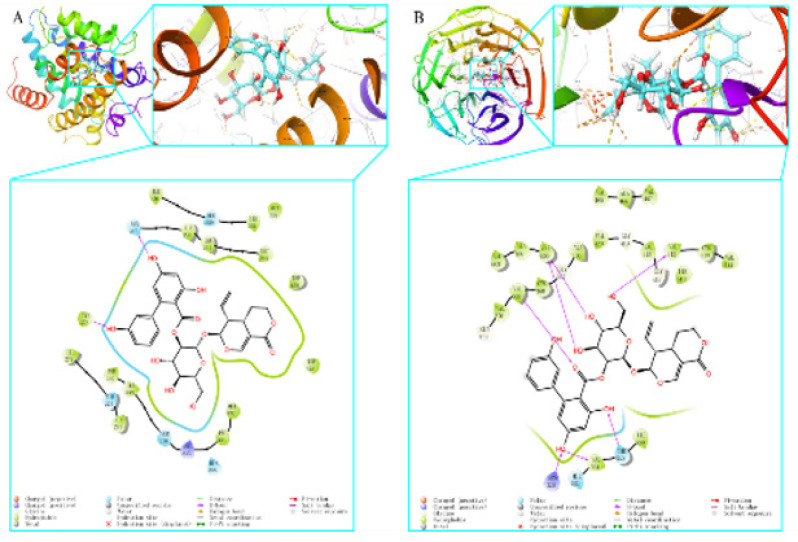


**Figure:**
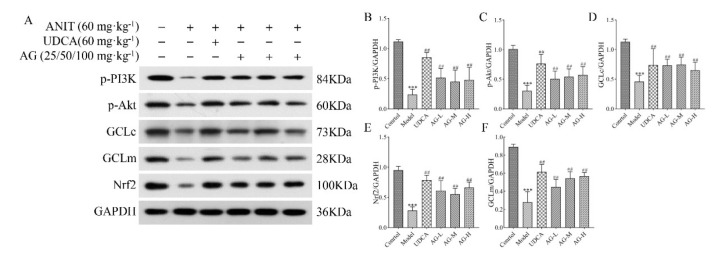


**Figure:**
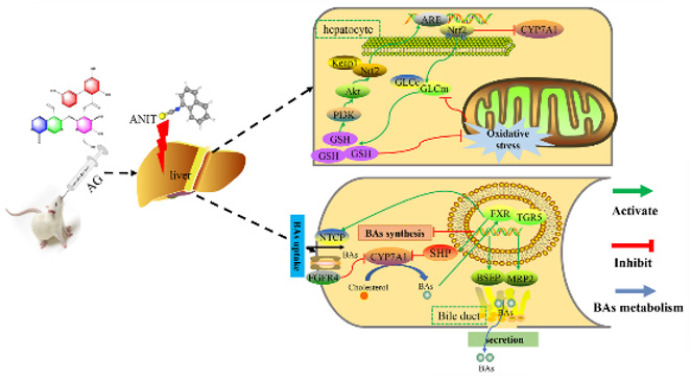


## Conclusion

AG regulated several metabolic enzymes and transporters related to bile acids by up-regulating the FXR and Nrf2 pathways, thus significantly reducing hepatic ANIT-induced injury. Therefore, potentiating the activities of FXR and Nrf2 could be a novel strategy for preventing and treating cholestasis. Additionally, AG could be considered a possible option for treating cholestasis. The protective properties of AG and natural medicines containing AG on ANIT-induced cholestasis should be further investigated, especially the molecular mechanism by which AG improves cholestasis. 

## Data Availability

Due to confidentiality concerns, the datasets created and/or analyzed in this study are not publicly accessible; however, they can be obtained from the corresponding author upon reasonable request.

## Declaration

We have not used any AI tools or technologies to prepare this manuscript.

