# Supplementary material for: Amarogentin relieves cholestatic liver injury caused by ANIT in rats by regulating the FXR and Nrf2 pathways
Source: Iran J Basic Med Sci. 2026;29(1):65–73. doi: 10.22038/ijbms.2025.87063.18815 (PMC12867094; doi:10.22038/ijbms.2025.87063.18815)
Supplement: Supplementary file 1 [file ijbms-29-1-65-s001.pdf]

The raw data of the Western Blots Images

FXR:

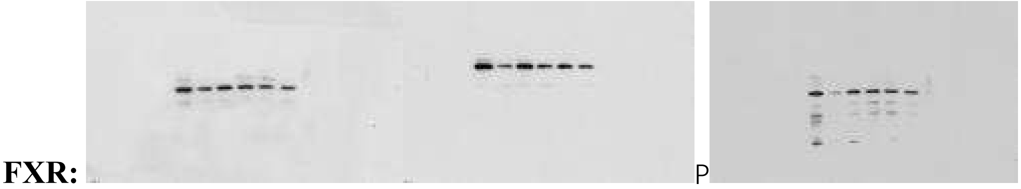

CYP7A1:

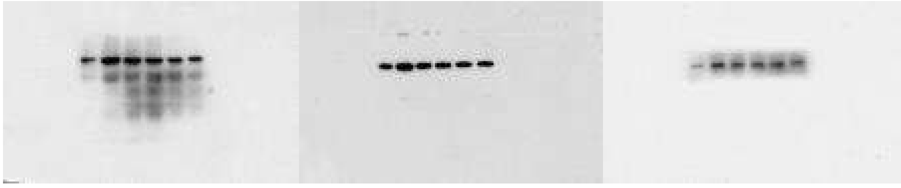

MRP2:

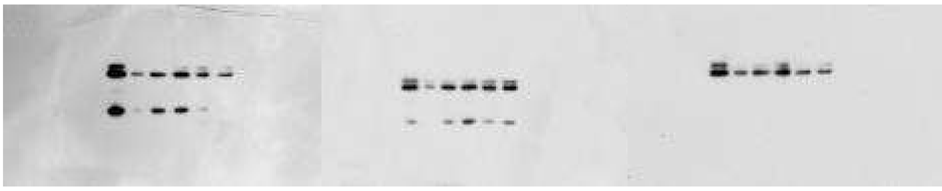

BSEP:

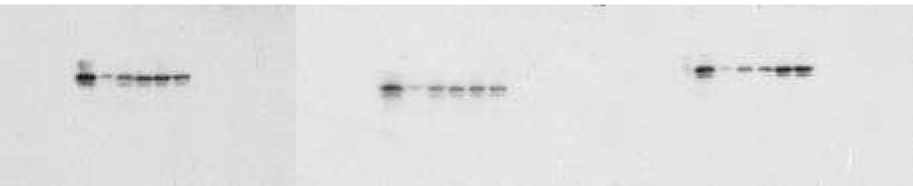

NTCP:

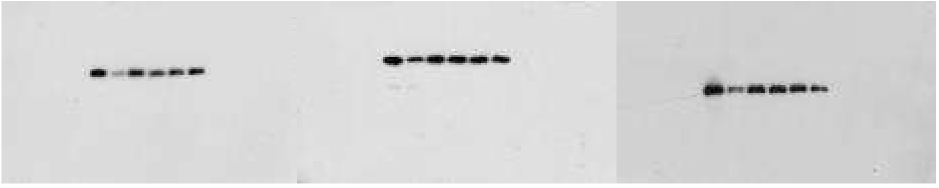

SHP:

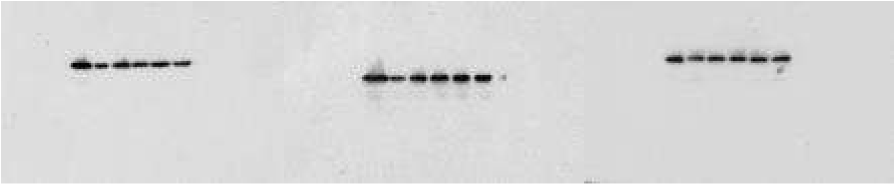

TGR5:

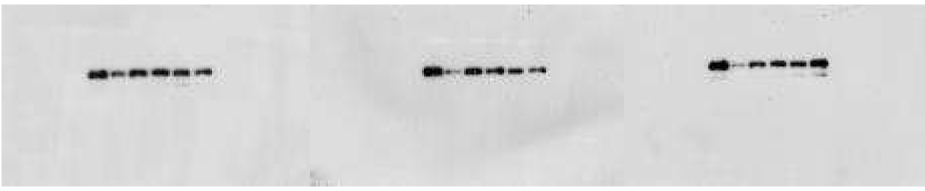

FGFR4:

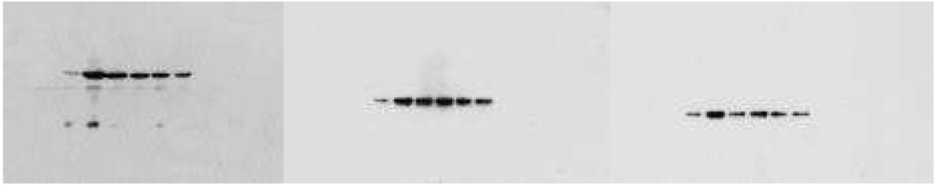

The raw data of the Western Blots Images in Figure 6

p-PI3K:

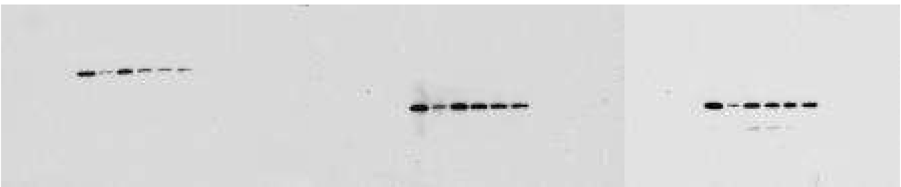

p-Akt:

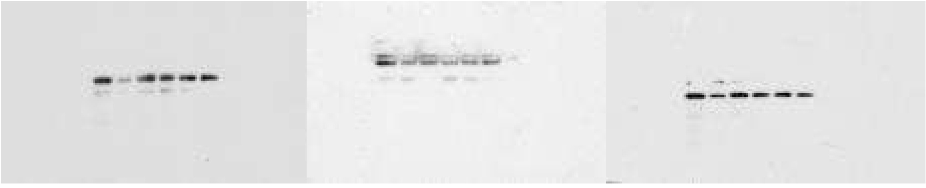

GCLc:

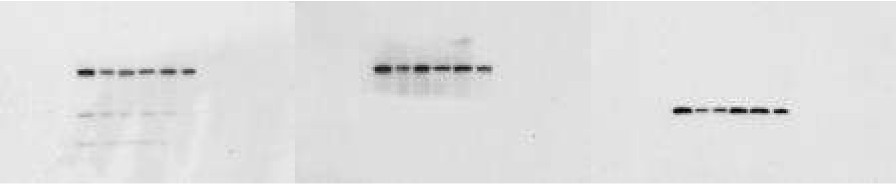

GCLm:

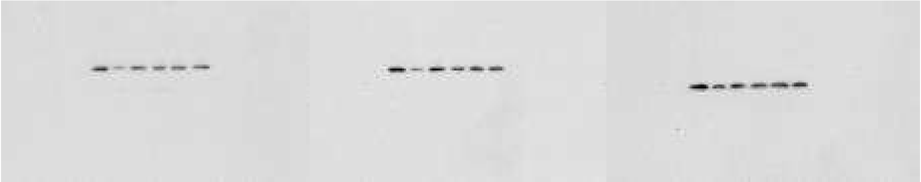

Nrf2:

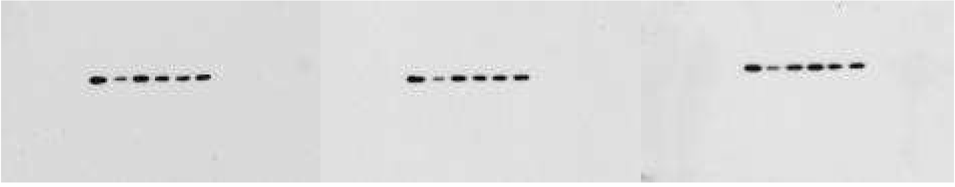

The raw data of the Western Blots Images in Figure 7
